# Phosphatase inhibitors with anti-angiogenic effect *in vitro*

**DOI:** 10.1111/j.1600-0463.2009.02561.x

**Published:** 2010-01

**Authors:** LENE SYLVEST, CHRISTINE DAM BENDIKSEN, GUNNAR HOUEN

**Affiliations:** Department of Clinical Biochemistry and Immunology, Statens Serum InstitutCopenhagen, Denmark

**Keywords:** Angiogenesis inhibition, ELISA-based screening assay, endothelial cells, phosphatase inhibitors, morphology

## Abstract

Sylvest L, Bendiksen CD, Houen G. Phosphatase inhibitors with anti-angiogenic effect *in vitro*. APMIS 2010; 118: 49–59.

Levamisole has previously been identified as an inhibitor of angiogenesis *in vitro* and *in vivo*, but the mechanism behind the anti-angiogenic behavior has not yet been established. However, one known effect of levamisole is the inhibition of alkaline phosphatase, and this fact encouraged us to test other phosphatase inhibitors for their anti-angiogenic effects by using the same method as used to identify levamisole: an ELISA-based co-culture angiogenesis assay giving quantitative and qualitative results. Historically, intracellular phosphatases have been associated with the downregulation of signaling pathways, and kinases with their upregulation, but lately, the phospatases have also been coupled to positive signaling, which is why inhibition of phosphatases has become associated with anti-tumorigenic and anti-angiogenic effects. The results obtained in this work reveal several agents with anti-angiogenic potential and give a strong indication that phosphatase inhibition is linked to anti-angiogenic activity. An apparent disruption of endothelial tube formation was seen for seven of eight phosphatase inhibitors tested in the angiogenesis assay. By looking at the morphological results, it was seen that most of the inhibitors impaired proliferation and elongation of the endothelial cells, which still had a differentiated appearance. One inhibitor, PTP inhibitor IV, seemed to impair endothelial cell differentiation and induced the same morphology as when cells were treated with levamisole, although at a 200 times lower concentration than that of levamisole. Hence, our work points out compounds with a potential that may be of use in the search for new medical products for the treatment of malignant tumors, or other conditions where angiogenesis plays a central role.

## Angiogenesis

To grow beyond the size of 1–2 mm^3^, a solid tumor depends on angiogenesis, the development of new blood vessels from pre-existing vasculature (1). In the adult, angiogenesis is tightly controlled by a balance between pro- and anti-angiogenic factors, and the vasculature is mainly quiescent (2), except during the female reproductive cycle, wound healing and in relation to pathological conditions such as cancer, rheumatoid arthritis and diabetic retinopathy (3). Pro- and anti-angiogenic factors can derive from cancer cells, endothelial cells, stromal cells, the bloodstream and the extracellular matrix (ECM). Angiogenesis involves the coordination of several steps: (i) binding of angiogenic stimulating factors [vascular endothelial growth factor (VEGF) and fibroblast growth factor (FGF)] to their receptors on endothelial cells, (ii) secretion of proteases, which degrade the vascular basement membrane (VBM) and the surrounding ECM, (iii) endothelial proliferation and migration, (iv) endothelial differentiation and tube formation, (v) reformation of the VBM, (vi) recruitment of pericytes, which surround and stabilize the newly formed vessel (4–6).

## Inhibition of angiogenesis

An effective approach to starving a tumor is to inhibit the development of new tumor blood vessels, and thereby reduce tumor progression. Several angiogenesis inhibitors combined with traditional cancer therapy have been shown to prolong the survival of patients with advanced cancer. A few of them are now in clinical use and effective toward some types of cancer (7), one of them being Bevacizumab (Avastin® Roche, Basel, Switzerland), a neutralizing antibody against VEGF (8). However, the inhibitors have been seen to cause serious side effects (9–11), and identification of new angiogenesis inhibitors with limited toxicity and a broader efficiency is still needed, as well as a further understanding of endothelial cell signaling to comprehend the mechanisms of action of potential inhibitors.

## Screening assay for identification of new potential angiogenesis inhibitors

An ELISA-based co-culture angiogenesis assay, with endothelial cells growing on a layer of fibroblasts, has previously been developed and serves as a screening assay for the identification of new potential anti-angiogenic agents (8). The endothelial cells form a capillary-like network when propagated for 3 days in co-culture with fibroblasts. The assay covers several steps in the angiogenic cascade unlike many previously established *in vitro* angiogenesis methods, which only measure a single step, and in addition to morphological results the assay provides quantitative ELISA results.

## Classification of angiogenesis inhibitors

Using the co-culture assay, well-known angiogenesis inhibitors representing different structural and functional classes could be divided into two groups according to their effect on endothelial cell morphology (12). The cells could be classified either as separate non-proliferating cells forming non-canalized short cords without interconnections or as undifferentiated non-elongated cells appearing as compact cell clusters. The group of inhibitors causing short cords of endothelial cells consisted of several known angiogenesis inhibitors, such as platelet factor 4 (PF4) (13), thrombospondin (TSP) (14), rapamycin (15), suramin (16), TNP-470 (17) and non-steroidal anti-inflammatory drugs (18). In general, they block proliferation, but their mechanisms are quite different from one another.

The other group of inhibitors, which caused cell clusters in the assay, are VEGF antibodies (7), a VEGF receptor tyrosine kinase inhibitor SU5614 (19) and the recently identified angiogenesis inhibitor levamisole (9, 20). Levamisole has also been shown to reduce tumor growth and angiogenesis in nude mice (20).

The mechanism behind the observed anti-angiogenic effect of levamisole remains unknown, but because of the very similar cell morphology induced by the three inhibitors in this group, they possibly block similar cellular signaling pathways and the effect of levamisole is very likely to be found in the pathways triggered by VEGF receptor binding. One of the known functions of levamisole is the inhibition of alkaline phosphatase (21), and this prompted us to test other phosphatase inhibitors in the assay.

## Materials and methods

### Chemicals, reagents, and cell lines

Ibandronate sodium salt, AP-conjugated goat anti-mouse IgG, 5-bromo-4-chloro-3-indolyl phosphate/nitro blue tetrazolium (BCIP/NBT) tablets, *p*-nitrophenyl phosphate (*p*-NPP) tablets, dimethyl sulfoxide (DMSO), levamisole, and bovine serum albumin were from Sigma-Aldrich (St. Louis, MO, USA). Clodronate disodium salt, salubrinal, Protein tyrosine phosphatase 1B (PTP1B) inhibitor, Shp1/2 inhibitor (NSC87877), PTP inhibitor I, II, and IV were from Merck (Darmstadt, Germany). MilliQ water equipment was from Millipore (Billerica, MA, USA). Mouse anti-human CD31 monoclonal antibody was from Monosan (Uden, The Netherlands). Alkaline substrate buffer, mouse anti-human double-stranded DNA antibody, PBS, and TTN buffer were from SSI Diagnostics (Hillerød, Denmark). Ethanol 96% was from De Danske Spritfabrikker (Aalborg, Denmark). HEPES buffer, trypsin/EDTA, trypsin neutralizing solution (TNS), human umbilical vein endothelial cells (HUVEC), normal human dermal fibroblasts (NHDF), HUVEC media-kit EGM-2 Bulletkit and NHDF media-kit FGM-2 Bulletkit were from LONZA (Basel, Switzerland). Recombinant human basic fibroblast growth factor (hFGF-B), and recombinant human VEGF were from R & D Systems (Minneapolis, MN, USA). Precision plus pre-stained molecular weight marker was from Bio-Rad (Hercules, CA, USA).

### Preparation of NHDF

NHDF were cultured in 75 cm^2^ culture flasks at 37 °C, 5% CO_2_ and 90% humidity in NHDF standard medium (FGM-2 Bulletkit) consisting of 100 ml fibroblast basal medium-2 (FBM-2) supplemented with 0.1 ml human basic fibroblast growth factor (hFGF-B), 0.1 ml insulin, 0.1 ml gentamicin/amphotericin (GA-1000) and 2% fetal bovine serum (FBS). Before harvesting the cells with trypsin/EDTA, they were washed 2 × 1 min with PBS. After a few minutes, trypsin/EDTA was neutralized with PBS. The suspension was centrifuged 10 min at 200 *g* and the pellet was resuspended in a known volume of FBM-2 medium before counting. Cells were seeded in a 96-microwell plate with 10^3^ cells in 100 μl NHDF standard medium per well and incubated for 3 days.

### Preparation of HUVECs

HUVECs were cultured in 25 cm^2^ culture flasks at 37 °C, 5% CO_2_ and 90% humidity in HUVEC standard medium (EGM-2 Bulletkit) consisting of 100 ml endothelial basal medium-2 (EBM-2) supplemented with 0.1 ml ascorbic acid, 0.4 ml hFGF-B, 0.1 ml recombinant3 insulin-like growth factor (R^3^-IGF)-1, 0.1 ml GA-1000, 0.1 ml heparin, 0.1 ml human epidermal growth factor (hEGF), 0.1 ml VEGF, 0.04 ml hydrocortisone and 2% FBS. The cell was culture incubated until the cells reached 70–90% confluence after approximately 3 days. Before harvesting, the cells were washed 1 × 1 min with HEPES-BSS. Trypsin/EDTA was added to the cells and incubated for 2 min at 37 °C to promote the detachment of cells. Trypsin was neutralized with TNS and the suspension was centrifuged for 5 min at 200 *g*. The pellet was resuspended in a known volume of EBM-2 medium before counting and the cells were now ready to be seeded on top of the fibroblasts in the microwell plate (see next section).

### Co-culture of NHDF and HUVEC

A previously published angiogenesis assay was performed essentially as described by Friis et al. (12, 22) and outlined briefly below. After 3 days of incubation in the 96-microwell plate, the fibroblasts had created a dense layer at the bottom of each well. The medium was removed and on top of the fibroblasts 10^3^ endothelial cells were added to each well in 135 μl TFSM2 + 10% (a reduced medium consisting of 100 ml EBM-2 medium supplemented only with 0.11 ml ascorbic acid, 0.11 ml GA-1000, 0.11 ml hEGF and 2.2% FBS) with either the presence of 15 μl phosphatase inhibitor in different concentrations, 15 μl phosphatase inhibitor solvent only (milliQ water or DMSO) or 15 μl EBM-2 medium only. After 3 days of co-culture, the ELISA could be performed.

### ELISA on co-culture cells

The medium was removed from the plate after 3 days, and the cells were washed for 1 min with PBS, 200 μl/well, followed by fixation with 100 μl/well 96% ethanol (−20 °C) for 15 min. All further incubations were performed at room temperature using 200 μl/well for washing and blocking unless otherwise stated. The cells were washed 2 × 1 min with TTN buffer followed by blocking of non-specific binding for 1 h with the same buffer. Primary antibodies were diluted in TTN buffer and plates were incubated either at 4 °C overnight or for 1 h at room temperature with 100 μl/well of either mouse anti-human dsDNA (1:20 000), mouse anti-human CD31 (1:250) or TTN buffer, followed by washing 3 × 1 min with TTN. 100 μl AP-conjugated goat anti-mouse IgG (1:1000) was added to all the wells and incubated for 1 h and again the wells were washed 3 × 1 min with TTN. To quantify the detected antigen, 100 μl 1 mg/ml *p*-NPP dissolved in alkaline substrate buffer with 1 mM levamisole (as an inhibitor of endogenous AP) was added to each well. The absorbance was read after 30, 60 and 120 min at 405 nm with background subtraction at 650 nm using a Versamax plate reader (MDC, Sunnyvale, CA, USA). By definition, a compound acts as a specific angiogenesis inhibitor if the relative absorbance (absorbance measured with inhibitor present in medium in relation to absorbance measured with no inhibitor, but with the presence of inhibitor solvent in the medium) is at the same time below 0.8 for detection of CD31 and above 0.8 for detection of dsDNA. The definition was based on results obtained in the co-culture assay with well-known angiogenesis inhibitors and the fact that the measurement of dsDNA indicates the number of fibroblasts (23).

### Immunostaining of co-culture cells

After ELISA readings, the wells were washed 3 × 1 min with TTN followed by development for 1 h in 1 mg/ml BCIP/NBT dissolved in milliQ water. The wells were washed 2 × 1 min and further stored with milliQ water. The BCIP/NBT staining was inspected in an Olympus (Tokyo, Japan) IX 70 microscope and photographed with an Olympus DP12 digital camera at ×40 magnification.

## Results

The aim of the present work was to screen different phosphatase inhibitors for their effect on endothelial cell network formation, and thereby identify new potential anti-angiogenic compounds. The tested inhibitors are listed in Table 1 together with previously reported cellular effects and the phosphatases they most potently inhibit. The inhibitors represent a broad spectrum and were chosen according to availability and assumed targets. The effects of the inhibitors were analyzed using an assay with the advantage of gaining morphological results and quantitative ELISA results from the same well. The ELISA results reveal the inhibitor’s effect on cell number, and combining them with the morphological staining results gives details on how the cells have been affected (proliferation, differentiation and elongation).

**Table 1:** Phosphatase inhibitors tested in the assay listed along with their chemical structure, their reported effects and the phosphatases they inhibit

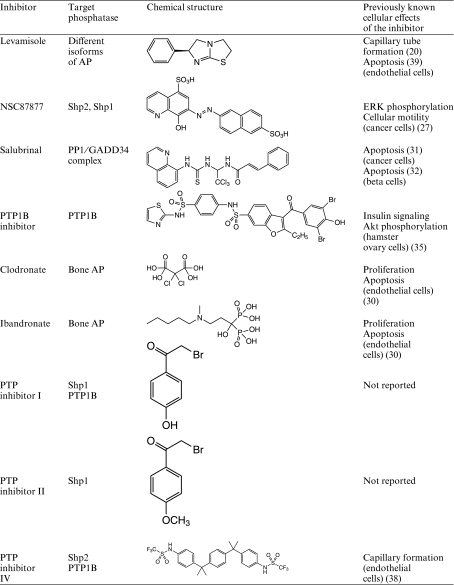

The endothelial cells were detected using CD31 antibody after 3 days of incubation in co-culture with or without phosphatase inhibitors, and for the detection of all cells present in the well (NHDF and HUVEC), an antibody against dsDNA was used. In the wells, the endothelial cells account for only around 1% of the total number of cells present, therefore dsDNA staining is actually a reflection of the number of fibroblasts in the well. The dsDNA is thereby used to determine if the inhibitors have a toxic effect on the fibroblasts.

The screening identified several compounds with anti-angiogenic potential. A suitable concentration range for each compound was determined by preliminary experiments and thereafter, an IC_50_ value (the concentration of the phosphatase inhibitor when 50% of the endothelial cell network formation had been inhibited compared to control) was calculated (Table 2). The obtained IC_50_ values extend over a large range of concentrations. The most effective of the tested compounds (with the lowest IC_50_) were PTP inhibitor I and PTP inhibitor II, both with an IC_50_ value of 3.7 μM and the compound with the highest IC_50_ was clodronate (800 μM). The PTP1B inhibitor was the only tested phosphatase inhibitor which had no effect on the cells in the angiogenesis assay. A few of the inhibitors had a toxic effect on the fibroblasts, which indicates that they are not endothelial cell specific and will be less suitable candidates for an anti-angiogenic product. These were ibandronate at concentrations ≥100 μM, PTPi I ≥ 10 μM, and PTPi II ≥100 μM.

**Table 2:** Results obtained when HUVECs were treated with phosphatase inhibitors in the co-culture angiogenesis assay

Inhibitor	IC_50_	Number of tests	Cell morphology
Levamisole	ND	2 (n = 4)	Clusters (2 mM)
NSC87877	0.23 ± 0.06 mM	4 (n = 8)	Short cords (0.2 mM)
Salubrinal	42 ± 9 μM^1^	3 (n = 6)	Short cords (20 μM)
PTP1B inhibitor	No inhibition	2 (n = 4)	Normal network at all tested concentrations
Ibandronate	ND	3 (n = 6)	Short cords/clusters (1 μM, 10 μM and 0.1 mM)
Clodronate	0.8 ± 0.2 mM	3 (n = 6)	Short cords (1 mM)
PTP inhibitor I	3.7 ± 0.2 μM	2 (n = 4)	Dead cells (10 μM)
PTP inhibitor II	3.7 ± 0.1 μM	2 (n = 4)	Dead cells (10 μM)
PTP inhibitor IV	40 ± 15 μM^1^	3 (n = 6)	Clusters (10 μM)

The concentration causing 50% inhibition of endothelial cell network formation (IC_50_) has been calculated from two to four independent tests, each time in duplicate (n = 4–8), and is illustrated in the Table ±SD. ND, not determined. The visualized cell morphology is stated along with the concentrations where the stated cell morphology is observed.

1 IC_50_ was found by extrapolation.

**Fig. 1:**
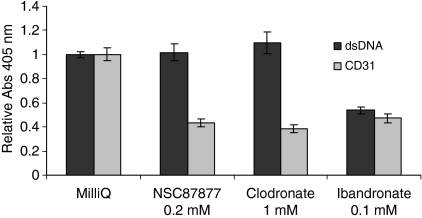
The phosphatase inhibitors NSC87877, clodronate and ibandronate impair HUVEC proliferation. ELISA results showing the inhibitory effect of 0.2 mM NSC87877, 1 mM clodronate and 0.1 mM ibandronate on HUVEC (CD31) and NHDF (dsDNA) grown in co-culture. Data are presented as relative absorbance (tested compound/milliQ water), from three to four independent experiments ± SD. Each experiment was performed in duplicate, n = 6–8.

The ELISA results for all the tested compounds are illustrated as relative absorbances compared to control in Figs. 1 and 3. The effect of phosphatase inhibitors dissolved in milliQ water is shown in Fig. 1 and is normalized to a control where 10% milliQ water was added to the medium instead of 10% phosphatase inhibitor. All the inhibitors had a strong reducing effect on the endothelial cell proliferation (CD31) with a relative absorbance around 0.4, hence below 0.8, which is the limit according to earlier findings regarding the inhibitory effect on the endothelial cells (23). The relative absorbance measured with dsDNA antibody, indicating the proliferation of fibroblasts, shows that NSC87877 and clodronate do not affect the fibroblasts (relative absorbance higher than 0.8). Ibandronate, however, has the same inhibitory effect on the fibroblasts as on the endothelial cells.

**Fig. 2:**
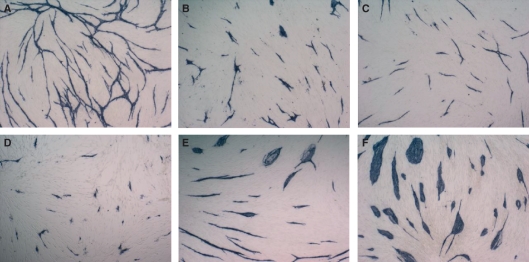
Immunostaining with CD31 antibody visualized the effects of NSC87877, clodronate and ibandronate on the endothelial cell network formation. Cells were treated with (A) milliQ water (control), (B) 0.2 mM NSC87877, (C) 1 mM clodronate, (D) 100 μM ibandronate, (E) 1 μM ibandronate and (F) 2 mM levamisole.

Looking at the morphological results obtained from CD31 immunostaining, NSC87877 and clodronate induce the endothelial cells to obtain short cord morphology without interconnections (Fig. 2B,C) compared to the control (Fig. 2A). The cells have a differentiated shape, but are not elongated as seen in the control. The morphology obtained is very different from the cell cluster morphology seen when 2 mM levamisole is present (Fig. 2F), which indicates that these phosphatase inhibitors use an anti-angiogenic mechanism different from that of levamisole and VEGF antibody. Ibandronate gave mixed morphological results depending on its concentration; 1 μM induced clusters and 100 μM induced short cords (Fig. 2D,E).

**Fig. 3:**
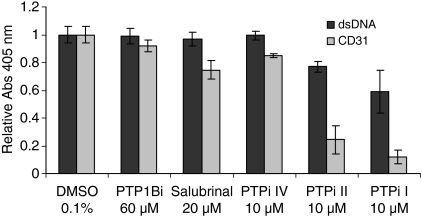
Effects of the PTP1B inhibitor, salubrinal, PTPi IV, PTPi II and PTPi I in the co-culture assay. ELISA results are illustrated when HUVEC (CD31) and NHDF (dsDNA) were treated with DMSO 0.1% (control), 60 μM PTP1B inhibitor, 20 μM salubrinal, 10 μM PTPi IV, 10 μM PTPi II and 10 μM PTPi I. Data are presented as relative absorbance (tested compound/milliQ water) from two to three independent experiments ± SD. Each experiment was performed in duplicate, n = 4–6.

**Fig. 4:**
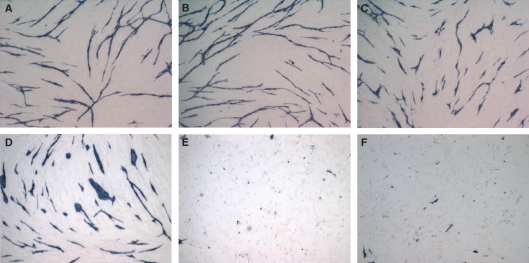
Immunostaining with CD31 antibody visualized the effects of the PTP1B inhibitor, salubrinal, PTPi IV, PTPi II and PTPi I on the endothelial cell network formation. Cells were treated with (A) DMSO 0.1% (control), (B) 60 μM PTP1B inhibitor, (C) 10 μM PTPi IV, (D) 20 μM salubrinal, (E) 10 μM PTPi II and (F) 10 μM PTPi I.

In Fig. 3 the effects of the phosphatase inhibitors dissolved in 0.1% DMSO are illustrated as relative absorbances normalized to control (0.1% DMSO). The PTP1B inhibitor is shown in the highest tested concentration (a higher concentration was not possible because it would result in a too high DMSO concentration), and no inhibitory effect on the cells is detected as both CD31 and dsDNA are higher than 0.8. The CD31 immunostaining results confirm that the PTP1B inhibitor has no effect on the cells (Fig. 4B) compared to the control (Fig. 4A). PTPi IV also has both CD31 and dsDNA above 0.8, but when compared with the morphological results (Fig. 4D), it is observed that the inhibitor had an inhibitory effect on the endothelial cells, despite the high CD31 detection in the ELISA procedure. The reason for this is that under these circumstances, the cells form multicellular clusters (same morphology as levamisole and VEGF antibody), which can result in a misleading high absorbance as the proliferation is not affected. This shows how important it is to compare the quantitative ELISA results with the morphological/qualitative results. This comparison has been made systematically with all the tested compounds regarding the detection of both dsDNA and CD31.

Salubrinal only had a minor effect on the endothelial cells at the highest possible concentration (20 μM) according to the ELISA results (Fig. 3). The immunostaining results show that the minor inhibitory effect was on elongation, as the cells are detected as short differentiated cords without interconnections (Fig. 4C). The compounds, PTPi II and PTPi I, have a very effective inhibitory effect on the endothelial cells at 10 μM. The endothelial cells treated with these compounds are almost absent (Fig. 4E,F), which indicates a rather toxic effect. Especially, PTPi I also has an inhibitory/toxic effect on the fibroblasts. Which endothelial cell morphology these compounds induce is difficult to determine because either they almost erase the cells or they have no effect on the normal network.

## Discussion

The work covered in this article describes different phosphatase inhibitors (Table 1) that were assayed for the effect on endothelial cell network formation in an *in vitro* co-culture angiogenesis assay. The background for testing phosphatase inhibitors was the identification of the anti-angiogenic activity of the AP-inhibitor levamisole (20). The coupling of anti-cancer and anti-angiogenic functions has previously been focused on the inhibition of kinases and thereby phosphorylation in cellular signaling pathways, but lately, the inhibition of phosphatases has also gained greater attention. The results obtained in this work reveal several potential anti-angiogenic agents, and give a strong indication that phosphatase inhibition is linked to anti-angiogenic activity because an obvious inhibition of endothelial tube formation was seen with seven of eight phosphatase inhibitors tested in the angiogenesis assay. In general, they influenced the cells to obtain the short cord morphology, which is an indication of blockage of endothelial cell proliferation, elongation and cell interconnections. Only PTPi IV induced distinct cell clusters, which is a sign of an inhibition of cell differentiation rather than proliferation. This is the morphology also seen when cells are treated with levamisole or VEGF antibody, and it indicates that PTPi IV has an effect in the pathways downstream of VEGFR2. Cell clusters were also seen with ibandronate treatment, but not to the same extent.

The endothelial cell morphology, which the phosphatase inhibitors induce, is also listed in Table 2, and in Table 1, earlier findings on cellular effect of the tested phosphatase inhibitors are noted briefly. These effects will be elaborated in the following section.

NSC87877 is a potent inhibitor of Shp2, a phosphatase known to promote several signaling pathways (22, 24–26). This inhibitor has previously been found by Chen et al. (27) to reduce viability of a breast cancer cell line, and they also detected an NSC87877 inhibition of EGF-induced Shp2 activity in these cells. Shp2 has also been coupled to VEGFR2 signaling pathways through Gab1 leading to Src activation resulting in increased migration and capillary formation (28). Testing NSC87877 in the co-culture angiogenesis assay revealed clear inhibition of the endothelial cell network development (IC_50_ = 0.23 mM). The individual cells had a differentiated appearance, but they were unable to make interconnections and were not elongated as untreated cells. NSC87877-induced endothelial cell inhibition resulted in a morphology very different from that of levamisole, which produces cell clusters, a morphology also seen when cells are treated with the VEGF antibody. However, it is still possible that NSC87877 affects the VEGF-induced signaling pathways, even though a morphology different from that with the VEGF antibody is obtained. VEGFR 2 binding activates a number of signaling pathways (29) and the antibody inhibits them all by blocking binding of VEGF to its receptor. The morphology seen with NSC87877-treated cells could result from only one or a few of the pathways being inhibited, possibly a component downstream of the VEGF receptor, e.g. Shp2.

The bisphosphonates, clodronate and ibandronate, also had a clear effect on the endothelial cells, although with a rather high IC_50_ of clodronate (800 μM). It was not possible to obtain an IC_50_ value of ibandronate because the results did not give a linear curve, but the immunostaining results clearly show that it is a very potent endothelial cell inhibitor, giving rise to the short cord morphology as well as to the cell cluster morphology. Fournier et al. (30) also made the observations that ibandronate and clodronate have an inhibitory effect towards angiogenesis, *in vitro* and *in vivo*, by inhibiting proliferation and inducing apoptosis. The endothelial cell inhibitory mechanisms of ibandronate and clodronate have still not been elucidated.

Salubrinal has been identified as an inhibitor of the PP1/GADD34 complex, which dephosphorylates eukaryotic translation initiation factor 2α (eIF2α). It has been connected with cell protective as well as pro-apoptotic functions in a cancer cell line (31) and in primary pancreatic beta-cells (32), respectively. Results in this work indicate that Salubrinal, at concentrations at 20 μM, has a minor inhibitory effect on the endothelial cells in the co-culture angiogenesis assay (IC_50_ = 42 μM, calculated by extrapolation), inducing a reduced endothelial network formation and short cord morphology. Whether the inhibitory effect is a result of apoptosis, as seen with the primary beta-cells, cannot be determined from our results, but the morphology obtained indicates inhibition of proliferation rather than a pro-apoptotic behavior.

The PTP1B inhibitor did not seem to have any inhibitory effect on the endothelial cells in the co-culture angiogenesis assay. However, the highest possible tested concentration was only 60 μM, because of the solvent DMSO, which would otherwise become too concentrated. Thus, whether the PTP1B inhibitor would inhibit the cells at a higher concentration is not known from this work. A previous study suggests that inhibiting PTP1B induces a pro-angiogenic effect, i.e. a promotion of endothelial cell proliferation and migration (33). In fibroblasts, however, PTP1B inhibition has been seen to result in a decreased cell migration (34). The specific PTP1B inhibitor used in this work has been seen to stimulate insulin signaling and Akt phosphorylation in ovary cells (35), but no references regarding effects on endothelial cells have been found.

PTPi I and PTPi II have both been shown to be potent inhibitors of Shp1, a phosphatase which inhibits VEGFR2 phosphorylation, and hence is a negative signaling component (36). However, the phosphatase inhibitors were the most effective angiogenesis inhibitors tested in this assay, indicating that they also inhibit a positive signaling component, or that Shp1 also has a positive effect on the cells. PTPi I and PTPi II also were shown to have a lethal and not only an inhibitory effect on the endothelial cells and fibroblasts (from 1 to 10 μM), which may make them less useful as a medical product.

In the assay, PTPi IV also turned out to be an efficient endothelial inhibitor, disturbing the network at 10 μM and inducing the cell cluster morphology as seen with levamisole and VEGF antibody. PTPi IV has been shown amongst others to be a potent inhibitor of Shp2 (37), as has also NSC87877. However, they induced different cell structures in the co-culture angiogenesis assay. Typically, the phosphatase inhibitors affect a large panel of different phosphatases. This could be the reason why we see different morphological results from PTPi IV and NSC87877. Also, previously, PTPi IV has been observed to impair capillary-like formation *in vitro* (38).

In conclusion, several phosphatase inhibitors were found to exert considerable angiogenesis inhibition *in vitro*. The inhibitory effects of such inhibitors will not be tumor vessel-specific, but can be expected to affect ongoing angiogenesis. Compared with other angiogenesis inhibitors like Avastin, small molecule inhibitors may offer some advantages. Avastin is administered as injections, whereas small molecule inhibitors may be taken orally. Avastin may give rise to antibody formation, and such antibodies may obliterate the therapeutic effect and give rise to adverse reactions. On the other hand, small molecule inhibitors may affect phosphatases and kinases in many different cell types and thus cause undesirable side reactions. The assay used here gives a preliminary evaluation of such effects, as the definition of angiogenesis inhibition in the assay relies on comparison of the relative effects on endothelial cells (HUVEC) and the general cytotoxic actions on fibroblasts (NHDF) and endothelial cells.

Summing up, the testing of a range of different phosphatase inhibitors clearly showed that amongst them are several anti-angiogenic agents, which may have the potential to be a new medical product. The clinically tolerable doses of most of the inhibitors are not known. An elucidation of their angiogenic inhibitory mechanisms and their effects *in vivo*, especially in comparison with VEGF antibodies, will be an obvious area for future research in the search for new clinically useful therapeutic anti-angiogenic drugs.

## References

[1] Folkman J (1990). What is the evidence that tumors are angiogenesis dependent?. J Natl Cancer Inst.

[2] Hanahan D, Folkman J (1996). Patterns and emerging mechanisms of the angiogenic switch during tumorigenesis. Cell.

[3] Carmeliet P, Jain RK (2000). Angiogenesis in cancer and other diseases. Nature.

[4] Klagsbrun M, Moses MA (1999). Molecular angiogenesis. Chem Biol.

[5] Papetti M, Herman IM (2002). Mechanisms of normal and tumor-derived angiogenesis. Am J Physiol Cell Physiol.

[6] Kalluri R (2003). Basement membranes: structure, assembly and role in tumour angiogenesis. Nat Rev Cancer.

[7] Folkman J (2006). Angiogenesis. Annu Rev Med.

[8] Ferrara N, Hillan KJ, Gerber HP, Novotny W (2004). Discovery and development of bevacizumab, an anti-VEGF antibody for treating cancer. Nat Rev Drug Discov.

[9] Elice F, Rodeghiero F, Falanga A, Rickles FR (2009). Thrombosis associated with angiogenesis inhibitors. Best Pract Res Clin Hematol.

[10] Nalluri SR, Chu D, Keresztes R, Zhu X, Wu S (2008). Risk of venous thromboembolism with the angiogenesis inhibitor Bevacizumab in cancer patients: a meta analysis. JAMA.

[11] Daher IN, Yeh ET (2008). Vascular complications of selected cancer therapies. Nat Pract Cardiovasc Med.

[12] Friis T, Hansen AB, Houen G, Engel AM (2006). Influence of angiogenesis inhibitors on endothelial cell morphology in vitro. APMIS.

[13] Bikfalvi A (2004). Platelet factor 4: an inhibitor of angiogenesis. Semin Thromb Hemost.

[14] Ren B, Yee KO, Lawler J, Khosravi-Far R (2006). Regulation of tumor angiogenesis by thrombospondin-1. Biochim Biophys Acta.

[15] Fasolo A, Sessa C (2008). mTOR inhibitors in the treatment of cancer. Expert Opin Invest Drugs.

[16] Zaniboni A (1990). Suramin: the discovery of an old anticancer drug. Med Oncol Tumor Pharmacother.

[17] Kruger EA, Figg WD (2000). TNP-470: an angiogenesis inhibitor in clinical development for cancer. Expert Opin Invest Drugs.

[18] Meric JB, Rottey S, Olaussen K, Soria JC, Khavat D, Rixe O (2006). Cyclooxygenase-2 as a target for anticancer drug development. Crit Rev Oncol Hematol.

[19] Spikermann K, Faber F, Voswinckel R, Hiddemann W (2002). The protein tyrosine kinase inhibitor SU5614 inhibits VEGF-induced endothelial cell sprouting and induces growth arrest and apoptosis by inhibition of c-kit in AML cells. Exp Hematol.

[20] Friis T, Engel AM, Klein BM, Rygaard J, Houen G (2005). Levamisole inhibits angiogenesis in vitro and tumor growth in vivo. Angiogenesis.

[21] Van Belle H (1976). Alkaline phosphatase. I. Kinetics and inhibition by levamisole of purified isoenzymes from humans. Clin Chem.

[22] Mohi MG, Neel BG (2007). The role of Shp2 (PTPN11) in cancer. Curr Opin Genet Dev.

[23] Friis T, Kjaer SB, Engel AM, Rygaard J, Houen G (2003). A quantitative ELISA-based co-culture angiogenesis and cell proliferation assay. APMIS.

[24] Ren Y, Meng S, Mei L, Zhao ZJ, Jove R, Wu J (2004). Roles of Gab1 and SHP2 in paxillin tyrosine dephosphorylation and Src activation in response to epidermal growth factor. J Biol Chem.

[25] Wu CJ, O’Rourke DM, Feng GS, Johnson GR, Wang Q, Greene MI (2001). The tyrosine phosphatase SHP-2 is required for mediating phosphatidylinositol 3-kinase/Akt activation by growth factors. Oncogene.

[26] Yu DH, Qu CK, Henegariu O, Lu X, Feng GS (1998). Protein-tyrosine phosphatase Shp-2 regulates cell spreading, migration, and focal adhesion. J Biol Chem.

[27] Chen L, Sung SS, Yip ML, Lawrence HR, Ren Y, Guida WC (2006). Discovery of a novel shp2 protein tyrosine phosphatase inhibitor. Mol Pharmacol.

[28] Laramee M, Chabot C, Cloutier M, Stenne R, Holgado-Madruga M, Wong AJ (2007). The scaffolding adapter Gab1 mediates vascular endothelial growth factor signaling and is required for endothelial cell migration and capillary formation. J Biol Chem.

[29] Ferrara N, Gerber HP, LeCouter J (2003). The biology of VEGF and its receptors. Nat Med.

[30] Fournier P, Boissier S, Filleur S, Guglielmi J, Cabon F, Colombel M (2002). Bisphosphonates inhibit angiogenesis in vitro and testosterone-stimulated vascular regrowth in the ventral prostate in castrated rats. Cancer Res.

[31] Boyce M, Bryant KF, Jousse C, Long K, Harding HP, Scheuner D (2005). A selective inhibitor of eIF2alpha dephosphorylation protects cells from ER stress. Science.

[32] Cnop M, Ladriere L, Hekerman P, Ortis F, Cardozo AK, Dogusan Z (2007). Selective inhibition of eukaryotic translation initiation factor 2 alpha dephosphorylation potentiates fatty acid-induced endoplasmic reticulum stress and causes pancreatic beta-cell dysfunction and apoptosis. J Biol Chem.

[33] Soeda S, Shimada T, Koyanagi S, Yokomatsub T, Muranob T, Shibuyab S (2002). An attempt to promote neo-vascularization by employing a newly synthesized inhibitor of protein tyrosine phosphatase. FEBS Lett.

[34] Liang F, Lee SY, Liang J, Lawrence DS, Zhang ZY (2005). The role of protein-tyrosine phosphatase 1B in integrin signaling. J Biol Chem.

[35] Wiesmann C, Barr KJ, Kung J (2004). Allosteric inhibition of protein tyrosine phosphatase 1B. Nat Struct Mol Biol.

[36] Guo D, Jia Q, Song HY, Warren RS, Donner DB (1995). Vascular endothelial cell growth factor promotes tyrosine phosphorylation of mediators of signal transduction that contain SH2 domains. Association with endothelial cell proliferation. J Biol Chem.

[37] Huang P, Ramphal J, Wei J, Liang C, Jallal B, McMahon G (2003). Structure-based design and discovery of novel inhibitors of protein tyrosine phosphatases. Bioorg Med Chem.

[38] Mannell H, Hellwig N, Gloe T, Plank C, Sohn HY, Groesser L (2008). Inhibition of the tyrosine phosphatase SHP-2 suppresses angiogenesis in vitro and in vivo. J Vasc Res.

[39] Artwohl M, Holzenbein T, Wagner L, Freudenthaler A, Waldhausl W, Baumgartner-Parzer SM (2000). Levamisole induced apoptosis in cultured vascular endothelial cells. Br J Pharmacol.

